# Functional Disability on ADL and IADL Among Rural versus Frontier Older Adults in Wyoming

**DOI:** 10.1093/geroni/igaf122.2768

**Published:** 2025-12-31

**Authors:** Nancy Karlin

**Affiliations:** University of Northern Colorado, Greeley, Colorado, United States

## Abstract

Given the unique challenges faced by frontier aging older adults, such as limited access to healthcare and social services, the potential for different functional disabilities as compared to rural older adults exists. A total of 142 community-dwelling older adults participated with 72 frontier and 70 rural county respondents. Participant sites were identified through the Division of Aging in Wyoming. The survey consisted of demographic questions, the Katz ADL Index, and Lawton Brody’s IADL Scale. The aim of the study was to identify similarities and differences between the two cohorts on ADL and IADL functional disability along with respondent characteristics. A chi-square test of independence showed no significant association between rural and frontier respondents and the number of ADL disabilities reported, X2 (4, N = 142) = 2.18, p = .70, or in the number of IADL functional disabilities, X2 (6, N = 142) = 2.67, p = .84. A larger number of frontier residing older adults reported being widowed than rural older respondents. No significant association was evident between rural and frontier respondents and who they lived with at the time of survey, X2 (4, N = 142) = 2.77, p = .59. However, those in frontier counties were more likely to be living alone with limited ADL functional disparity, X2 (8, N = 142) = 21.9, p < .005. Rural older adults reported higher levels of education associated with few functional disabilities. These and additional findings offer insight into the aging experiences of both groups.

